# Supplementary material for: Genome-centric analysis of short and long read metagenomes reveals uncharacterized microbiome diversity in Southeast Asians
Source: Nat Commun. 2022 Oct 13;13:6044. doi: 10.1038/s41467-022-33782-z (PMC9561172; doi:10.1038/s41467-022-33782-z)
Supplement: Supplementary file 3 — Description of Additional Supplementary Files [file 41467_2022_33782_MOESM3_ESM.docx]

**Genome-centric analysis of short and long read metagenomes reveals uncharacterized microbiome diversity in Southeast Asians**

**Description of Additional Supplementary Files**

**File name:** Supplementary Data 1

**Description:** SPMP sample metadata.

**File name:** Supplementary Data 2

**Description:** Sequencing statistics.

**File name:** Supplementary Data 3

**Description:** Statistics for Metagenome Assembled genomes (MAGs) for both hybrid-, short-read and Hi-C binned MAGs.

**File name:** Supplementary Data 4

**Description:** Genome Taxonomy Database (GTDB) accession numbers of both isolate and uncultivated species genomes whose qualities where improved from medium to high with hybrid MAGs in this study, and associated metadata.

**File name:** Supplementary Data 5

**Description:** MaAsLin2 statistical associations between genera and demographic factors.

**File name:** Supplementary Data 6

**Description:** Taxonomic assignments of MAGs in this study to Human Reference Gut Microbiome (HRGM) genomes based on Mash distances.
