# Supplementary material for: Genome-centric analysis of short and long read metagenomes reveals uncharacterized microbiome diversity in Southeast Asians
Source: Nat Commun. 2022 Oct 13;13:6044. doi: 10.1038/s41467-022-33782-z (PMC9561172; doi:10.1038/s41467-022-33782-z)
Supplement: Supplementary file 10 — Reporting Summary [file 41467_2022_33782_MOESM10_ESM.pdf]

nature portfolio

Corresponding author(s): Niranjan Nagarajan
Henning Seedorf
Teo Yik-Ying

Last updated by author(s): Sep 9, 2022

Reporting Summary

Nature Portfolio wishes to improve the reproducibility of the work that we publish. This form provides structure for consistency and transparency in reporting. For further information on Nature Portfolio policies, see our Editorial Policies and the Editorial Policy Checklist.

Statistics

For all statistical analyses, confirm that the following items are present in the figure legend, table legend, main text, or Methods section.

n/a	Confirmed	
☐	☒	The exact sample size (n) for each experimental group/condition, given as a discrete number and unit of measurement
☒	☐	A statement on whether measurements were taken from distinct samples or whether the same sample was measured repeatedly
☐	☒	The statistical test(s) used AND whether they are one- or two-sided<br>*Only common tests should be described solely by name; describe more complex techniques in the Methods section.*
☒	☐	A description of all covariates tested
☐	☒	A description of any assumptions or corrections, such as tests of normality and adjustment for multiple comparisons
☐	☒	A full description of the statistical parameters including central tendency (e.g. means) or other basic estimates (e.g. regression coefficient) AND variation (e.g. standard deviation) or associated estimates of uncertainty (e.g. confidence intervals)
☐	☒	For null hypothesis testing, the test statistic (e.g. F, t, r) with confidence intervals, effect sizes, degrees of freedom and P value noted<br>*Give P values as exact values whenever suitable.*
☒	☐	For Bayesian analysis, information on the choice of priors and Markov chain Monte Carlo settings
☒	☐	For hierarchical and complex designs, identification of the appropriate level for tests and full reporting of outcomes
☐	☒	Estimates of effect sizes (e.g. Cohen's d, Pearson's r), indicating how they were calculated

Our web collection on statistics for biologists contains articles on many of the points above.

Software and code

Policy information about availability of computer code

Data collection	Nanopore signal data was basecalled with the latest version of the basecaller available at the point of sequencing (Guppy v3.0.4 to v3.2.6). Basecalled nanopore reads were demultiplexed and filtered for adapters with qcat (v1.1.0).
Data analysis	The following software packages/libraries were used: Fastq-Scan (v0.4.1), NanoStat (v1.4.0), Kraken (v2.1.1), Bracken (v2.6.1, v2.6.0), MEGAHIT (v1.04), OPERA-MS (v0.9.0), MetaBAT2 (v2.12.1), CheckM (v1.04), Barnnap (v0.9), tRNAscan-SE (v2.0.5), GUNC (v10.4), Prodigal (v2.6.3), GTDBtk (v1.4.1), Mash (v2.3), sklearn (v0.32.2), biopython (v1.78), FastANI (v1.32), FigTree (v1.4.4), iNEXT (v.2.1.7), MaAsLin2 (v1.4v0), minimap2 (vv2.24-r1122), bamtools (v2.5.2), samtools fastq (v1.15.1), pysam (v0.19.1), antiSMASH (v5.1.2), DeepBGC (v0.1.18), BiG-SCAPE (v1.01), Clinker (v0.0.12), Fastspar (v1.0.0), AMPscanner (v2.0), AmpGram (v1.0), AMPDiscover (v1.0), ABPDiscover (v1.0), seqkit (v0.11.0), samtools faidx (v1.9).<br><br>Custom code used for this study is available on GitHub at https://github.com/CSB5/SPMP.

For manuscripts utilizing custom algorithms or software that are central to the research but not yet described in published literature, software must be made available to editors and reviewers. We strongly encourage code deposition in a community repository (e.g. GitHub). See the Nature Portfolio guidelines for submitting code & software for further information.

Data

Policy information about availability of data

All manuscripts must include a data availability statement. This statement should provide the following information, where applicable:

- Accession codes, unique identifiers, or web links for publicly available datasets
- A description of any restrictions on data availability
- For clinical datasets or third party data, please ensure that the statement adheres to our policy

Source data are provided with this paper. Shotgun metagenomic sequencing data (Illumina and ONT) and SPMP hybrid MAGs are available from the European Nucleotide Archive (ENA – https://www.ebi.ac.uk/ena/browser/home) under project accession number PRJEB49168. All SPMP MAGs, a corresponding Kraken database, gene annotations and BGC sequences are available on Figshare at https://figshare.com/collections/SPMP/5993596.

SPMP genomes were compared to the GTDB database (release 95, https://gtdb.ecogenomic.org). UHGG genomes and a corresponding Kraken database are available from http://ftp.ebi.ac.uk/pub/databases/metagenomics/mgnify_genomes/human-gut/v1.0/. SGB genomes are available from http://segatalab.cibio.unitn.it/data/Pasolli_et_al.html. HRGM genomes from the initial release are available from https://www.mbiomenet.org/HRGM/. The Kraken standard database used to assess Bifidobacterium abundances is available from https://benlangmead.github.io/aws-indexes/k2. Databases used for antiSMASH analysis of SPMP BGCs are available from v5.1.2 of the antiSMASH software, while the MIBiG 2.0 database are available from https://mibig.secondarymetabolites.org/download.

Source code for scripts used to analyze the data are available in a GitHub project at https://github.com/CSB5/SPMP.

Field-specific reporting

Please select the one below that is the best fit for your research. If you are not sure, read the appropriate sections before making your selection.

☒ Life sciences ☐ Behavioural & social sciences ☐ Ecological, evolutionary & environmental sciences

For a reference copy of the document with all sections, see nature.com/documents/nr-reporting-summary-flat.pdf

Life sciences study design

All studies must disclose on these points even when the disclosure is negative.

Sample size	Sample size was determined based on available resources, as such no statistical analysis was used to predetermine sample size.
Data exclusions	No data was excluded in this study.
Replication	This is cross sectional study without sample replicate.
Randomization	No randomization was required as the study design was observational.
Blinding	No blinding was planned as the study design was observational.

Reporting for specific materials, systems and methods

We require information from authors about some types of materials, experimental systems and methods used in many studies. Here, indicate whether each material, system or method listed is relevant to your study. If you are not sure if a list item applies to your research, read the appropriate section before selecting a response.

Materials & experimental systems

n/a	Involved in the study	
☒	☐	Antibodies
☒	☐	Eukaryotic cell lines
☒	☐	Palaeontology and archaeology
☒	☐	Animals and other organisms
☐	☒	Human research participants
☒	☐	Clinical data
☒	☐	Dual use research of concern

Methods

n/a	Involved in the study	
☒	☐	ChIP-seq
☒	☐	Flow cytometry
☒	☐	MRI-based neuroimaging

Human research participants

Policy information about studies involving human research participants

Population characteristics	A complete description of the study participants can be found in Table S1. Cohort is composed of 109 healthy Singaporean. Age ranges from 48 to 76 years old with 65 females and 44 males. Population is divided in 3 ethnicities: 53 Chinese, 30 Indians and 26 Malays.
Recruitment	Subjects for this cross-sectional study were recruited based on recall from a community-based multi-ethnic prospective cohort that is part of the Singapore Population Health Studies project (SPHS -formerly Singapore Consortium of Cohort Studies). This subset included 109 subjects who were 48 to 76 years old with 65 males and 44 females. Subjects in SPHS were recruited to participate in the National Health Survey, where subjects were selected at random using age- and gender-stratified sampling to obtain a representative sample set of residents in the country. During the period of recruitment from April 16th, 2008 to September 20th, 2018 , subjects did not have any pre-existing major health conditions (cardiovascular disease, mental illness, diabetes, stroke, renal failure, hypertension and cancer) based on self-reporting. The ethnicity of each subject was confirmed verbally so that all four grandparents of the subject belonged to the same ethnic group. As such, we do not anticipate that any self-selection bias was introduced. A separate comparison of baseline clinical measurements was performed, including age-adjusted BMI and HbA1c, against the rest of the subjects in the larger ethnicity-specific cohorts within Singapore Population Health Studies to ensure that the sampling for the initial cohort conformed to population norms. Informed consent was obtained from all participants. Each subject was given 60 Singapore Dollars for their participation in this study. All associated protocols for this study were approved by the National University of Singapore Institutional Review Board (IRB reference number H-17-026) on May 9th, 2017 and renewed until May 31st, 2021.
Ethics oversight	Informed consent was obtained from all participants and the associated protocols for this study were approved by the National University of Singapore Institutional Review Board (IRB reference number H-17-026).

Note that full information on the approval of the study protocol must also be provided in the manuscript.
